# Addressing the Data Acquisition Paradigm in the Early Detection of Pediatric Foot Deformities

**DOI:** 10.3390/s21134422

**Published:** 2021-06-28

**Authors:** Paul D. Rosero-Montalvo, Edison A. Fuentes-Hernández, Manuel E. Morocho-Cayamcela, Luz M. Sierra-Martínez, Diego H. Peluffo-Ordóñez

**Affiliations:** 1 Department of Computer Science, IT University of Copenhagen, 2300 Copenhagen, Denmark; 2 Intelligence for Embedded Systems—Research Line, SDAS Research Group, Ibarra 100150, Ecuador; manuel.morocho@sdas-group.com (M.E.M.-C.); diego.peluffo@sdas-group.com (D.H.P.-O.); 3 Department of Technologies, Instituto Superior Tecnológico 17 de Julio, Urcuquí 100650, Ecuador; edicesc@gmail.com; 4 School of Mathematical and Computational Sciences, Yachay Tech University, Urcuquí 100650, Ecuador; 5 Systems Department, Faculty of Electronic Engineering and Telecommunications, Universidad del Cauca, Popayán 190001, Colombia; lsierra@unicauca.edu.co; 6 Department of Engineering, Corporación Universitaria Autónoma de Nariño, Pasto 520002, Colombia; 7 Modeling, Simulation and Data Analysis (MSDA) Research Program, Mohammed VI Polytechnic University, Benguerir 43152, Morocco

**Keywords:** children, plantar pressure, embedded systems, data analysis, machine learning

## Abstract

The analysis of plantar pressure through podometry has allowed analyzing and detecting different types of disorders and treatments in child patients. Early detection of an inadequate distribution of the patient’s weight can prevent serious injuries to the knees and lower spine. In this paper, an embedded system capable of detecting the presence of normal, flat, or arched footprints using resistive pressure sensors was proposed. For this purpose, both hardware- and software-related criteria were studied for an improved data acquisition through signal coupling and filtering processes. Subsequently, learning algorithms allowed us to estimate the type of footprint biomechanics in preschool and school children volunteers. As a result, the proposed algorithm achieved an overall classification accuracy of 97.2%. A flat feet share of 60% was encountered in a sample of 1000 preschool children. Similarly, flat feet were observed in 52% of a sample of 600 school children.

## 1. Introduction

The major functionality of the foot is to provide the necessary support and propulsion for the human body to move in a bipedal manner, carrying out different locomotive activities throughout the day. The study of foot bio-mechanic systems (FBSs) has allowed researchers to study and detect bad posture and the inadequate balance of the body [1,2,3]. Among the diverse research on FBSs, the analysis of foot sole pressure has become an essential detail for the proper diagnosis of problems in the lower spinal column, muscles, and joint injuries [4]. During physical rehabilitation, plantar pressure data provide crucial information for the planning of muscle recovery exercises [5]. Therefore, podometry is employed to study the distribution of plantar pressure and the relationship with the foot, ankle, knee, and hip during physical activities. This is based on the study of the anatomy of the foot, which allows describing its structure and parts: hindfoot, midfoot, forefoot, and the foot sole vault (providing resistance to the load, weight, and effort) [6].

**Figure 1:**
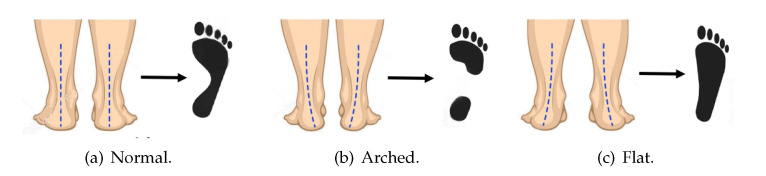
The three most common types of footprint shapes. (**a**) Normal, (**b**) arched, and (**c**) flat.

Several research efforts in the area of podometry have defined physiological and anatomical disorders related to human walking [6]. Among the main concerns, researchers have identified the use of different types of footwear, extreme forcing of the foot, and the limitations of flexion and extension [1]. The latter can be appreciated from the variations in the balance of the child’s foot, which generates, on the one hand, a concern for parents who receive sporadic medical consultations, which lead to aggressive treatments of the normal evolution of the foot, causing injuries and limiting the functional purpose of the joints. On the other hand, natural pathologies of the foot must be constantly monitored to avoid pain in physical activities, generating difficulties in the development of the mechanics of natural movements such as walking or jogging. For these reasons, establishing adequate footprint detection criteria and monitoring will enable children to correctly develop a functional locomotor system [7,8]. In this sense, the main pathological symptoms are, on the one hand, a pronated foot (i.e., flat foot), which is the absence of a foot sole vault, and, on the other hand, a supinated foot (i.e., arched foot), which corresponds to an increaseof the foot sole arch and presents an atypical elevation that causes the stiffening of the toes [9]. However, a footprint with an adequate weight distribution and balance is considered neutral, which does not represent any harm to the patient. Figure 1 depicts the different types of footprint shapes.

Currently, there are certain measurement instruments such as *podometers* and *podoscopes* that enable a medical analysis of foot sole pressure. The aforementioned systems can be employed in long-term patient monitoring; however, the presence of a medical specialist to interpret the data is always necessary to determine the patient’s condition [6]. This validation process has become a limitation for all kinds of massive studies of foot pathologies. As a case study, in Ecuador, forty-eight percent of the population is moderately overweight. Of this, one-point-five percent of women and 0.5% of men have cavus feet [10]. Furthermore, forty-five percent of women and 50% of men have flat feet. This high percentage is mainly due to the use of slippers in coastal areas [11]. For this reason, a deep study of children with the aim of correcting this problem early on is highly required, since the deformation of the footprint can be treated approximately until the age of 12 years. This notwithstanding, works such as [12,13] have mentioned that there is no such problem in children’s feet. However, if we take into account the signs of the wrong footprint in physical activities with unsuitable footwear, the child will not perform the mechanical process of walking and standing properly, leading to future problems [14,15].

Generally, the data collection process of a studied phenomenon is carried out by means of sensors, given that these electronic elements are built with transducers capable of converting a physical signal into an electrical one. For the latter process to be carried out without loss of information, an adequate coupling between the sensor and the system is required [16]. After the data acquisition process, it is possible to use the information to adapt any system to make its own decisions when learning from external impulses. For a system to be able to learn from external impulses, it is necessary to implement a supervised machine learning (ML) model with a robust training and validation dataset. Porting ML models into an independent embedded system (ES) has gained much attention from healthcare engineering researchers [17,18,19]. However, the learning stage is usually carried out on workstation computers and servers hosted in the cloud, which is not a unique solution since the response time, speed, and mobility require a great amount of computational resources. For these reasons, a computational paradigm was proposed to carry out these training processes locally (in the same ES [20]). As a result, intelligent systems can process their own information to generate a quick response, with an easy adaptation to any dynamic environment. The entire process is monitored by the validation system after the forecast is made by the ES [18]. This process was undertaken to enable an early detection system for locomotionproblems in preschool and school children in rural areas of the province of Imbabura-Ecuador, who currently do not have access to this information for free from educational and health centers. The main objective was to provide the teachers and health personnel of rural educational institutions an electronic system capable of constantly monitoring students without the need to pay for costly and lengthy procedures to detect plantar pressure problems.

This paper proposed an intelligent embedded system to classify the type of footprint of preschool (three to five years old) and school (nine to twelve years old) children as pronated, supinated, and normal. First, a vector of resistive pressure sensors (FSRs) was attached to each foot utilizing the prevailing sizes of the child population studied. Secondly, a sensor-coupling stage was added to the system using hardware and software criteria. Thirdly, a robust dataset was generated to feed the machine learning model. However, the computational capabilities of the embedded system were limited. For this reason, a prototype was deployed with the aim of reducing the training matrix size. Subsequently, a comparison and analysis of state-of-the-art supervised classification algorithms were conducted through different performance metrics. The leading classification model was chosen due to its compromise of good decision making, a reasonable response time, and a low computational cost. Note that, the obtained results were validated under the supervision of medical professionals, who compared our results with conventional foot sole techniques, such as the Hernandez-Corvo analysis [21]. To test our proposal in real-world conditions, we deployed our classifier on an embedded system, reaching an overall performance of 97.2% when running the *k*-NN algorithm over a reduced data matrix (i.e., a matrix generated by the previously carried out prototype selection stage). For comparison purposes, we employed a decision tree algorithm as a reference model, reaching a classification accuracy of 100%. Therefore, we can highlight that the proposed approach obtained an acceptable performance while not exceeding the embedded system’s processing capabilities.

The remainder of the paper is organized as follows: Section 2 presents the relevant research efforts in the field. Section 3 introduces the detailed design of the proposed data acquisition scheme, as well as the embedded system design. In Section 4, we analyze the results of the proposed system, starting from the generation of the database, the selection criteria for the embedded system prototype, and the supervised classification performance metrics. The results of the electronic system, data analysis, visualization, and tests in real conditions are presented in Section 5. Finally, Section 6 concludes the paper.

## 2. Related Works

The FBSs’ research community has been very active since the first sensors were built. Traditionally, capacitive, resistive, piezoelectric, and piezoresistive sensors have been implemented for the early diagnosis of lower spinal column problems. However, resistive sensors (RSs) have prevailed over their peers, in part due to their small size, low energy consumption, and minimal thickness [4]. Works, such as [1,5,22,23,24,25,26], have researched the detection of footfall through the use of embedded systems with FSRs. An FSR works by mapping the footprint using a vector of RSs. Other works, such as [27,28,29], have used several types of sensors in order to increase the precision of the data acquisition. However, the implementation cost increases significantly. All these works showed novel scientific-technological developments applying different electronic schemes for the correct acquisition of information. However, only [30] presented a supervised machine learning algorithm implementation to solve the problem of plantar pressure classification. The system in [30] could make its own decisions; however, it did not take into account the best representation of the studied phenomenon. For example, the system did not take into account the acquisition data stage (i.e., they did not generate their own dataset), and there was a lack of a visualization interface to record patient information and display the classifier inference.

In this paper, we tackled the aforementioned challenges as open research problems and introduced a low-complexity machine learning-based alternative system, able to detect foot sole problems at an early stage with a high prediction accuracy. Addressing this important problem can increase the quality-of-life of a large number of children with foot sole problems.

## 3. System Design

This section describes (a) the data acquisition hardware and its location in order to obtain the optimal plantar pressure data and (b) the signal preprocessing, coupling, and filtering (using software and the embedded system hardware) to generate the custom dataset.

### 3.1. The Impact of the Location of the Plantar Pressure Sensors

The factors influencing the distribution of the foot sole pressure are weight, age, and sex [10]. For this reason, we needed to establish functional parameters in the design of the system in order to have a homogeneous distribution of the foot’s weight without the use of shoes or insoles, which can introduce errors into the dataset [31].

In this work, two acquisition systems were designed to fulfill similar functions based on the anthropometric data obtained from the Ministry of Health of the Ecuadorian government (https://www.salud.gob.ec/ (accessed on 9 April 2021)) and the data provided by UNICEF (https://www.unicef.org/ecuador/media/3356/file/Encuesta%20Nacional%20de%20Salud%20y%20Nutrici%C3%B3n.pdf (accessed on 12 April 2021)), which contain data on the growth of children with physical parameters such as weight, height, body mass index, and body circumferences, which reflect, in a way, body composition. These parameters are of great importance for individual and collective health because they assess the general well-being of children. In general terms, preschoolers between three and five years old have a weight between 14.6 and 17.1 kg with a height between 94 and 102.8 cm with a variability of 5% between girls and boys. On the other hand, schoolchildren between nine and twelve years old have a weight between 21.4 and 37.4 kg and a height between 114.2 and 142.9 cm with a variability of 4% between boys and girls. For this reason, the sex of the child was not taken into account since the system detected a variety of foot sizes that met both criteria. We focused, on the one hand, on preschool children between three and five years old, with foot measurements between 15 and 16.5 cm and a width of 3.5 to 4.5 cm, and, on the other hand, on school children between nine and twelve years old, with foot a length between 20.2 and 22.4 cm and a width of 5.7 to 6.5 cm. This categorization was performed to segment the data at the beginning and end of childhood [10].

The dimensions were taken in relation to the sensor’s active area of 5 mm and the medianof the selected ages. After establishing the dimensions, we initiated the location of the sensors in order to obtain the most stable foot pressure values in relation to the types of footprint [1,5]. The latter foot zones are shown in Table 1. In addition, for the classification criteria, the corresponding labels were assigned after a deep analysis of the foot size and the greatest pressure points of the footprint.

**Figure 2:**
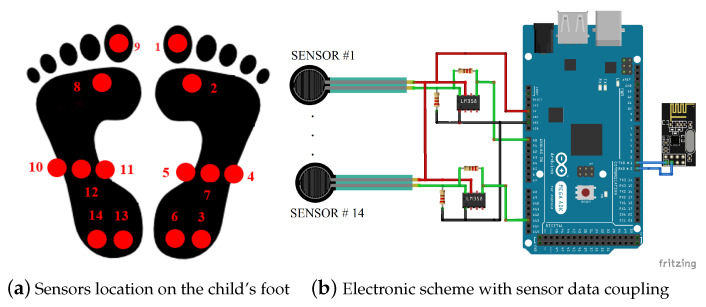
The proposed data acquisition system.

The Hernández-Corvo method was used to correctly establish the location of the sensors. This method allows recording different marksby setting the foot sole, covered with talc, on a black piece of cardboard [32]. This method establishes the location of the sensors at certain points: (a) first metatarsal, (b) union between the big toe and second toe, (c) middle half zone, (d) left end of the midfoot, (e) right side midfoot area, and (f) two sensors in the midheel area [33]. As a result, a total of 14 sensors were deployed for each type of child. We considered using the pressure system on both feet to have an accurate prognosis of the plantar pressure of each patient and capture slight variations in the weight distribution. In addition, the graphical results allowed the health personnel at the educational institutions to detect indications of the shortening of the lower extremities, which might have been undetected by most parents and health entities [8,13]. The distribution of the sensors and the connection method for their data acquisition can be seen in Figure 2.

### 3.2. Coupling and Filtering Sensor Data

For the footprint pressure sensors’ selection, the operating requirements were defined, the main ones being: reliability, precision, availability, ease-of-use, and scalability. As a result, the FSR 402 sensor was chosen for the system, because according to its datasheets, it generates an independent measurement of weight and pressure location. The proposed system also included a voltage divider to adjust the measured values, allowing configuring the amplification of the voltage in order to achieve high-input impedance to deliver an error-free value from the nonlinear electronic elements. This process was carried out with operational amplifiers (OAs). Equation (1) shows the gain of the OA with a high input impedance, where $R_{G}$ is the resistance that defines the gain factor and $R_{FSR}$ stands for the value of the internal resistance of the FSR.

(1) $$V_{out}=(\frac{R_{G}}{R_{FSR}}+1)V_{REF}.$$

When the force-sensitive resistor was pressed, the resistor $R_{F}SR$ limited the voltage gain in $V_{out}$ to 5 V. In this sense, $V_{REF}$ was also ±5 V to supply voltage to the electronic system.

In order to register the quantization stage error from the system (i.e., the analog–digital converter and the sensor coupling), two-hundred samples were acquired at a frequency of 1 kHz without pressure or active operation in the coupled sensor. To detect the frequency components of the noise introduced into the system, the fast Fourier transform (FFT) was employed as a preprocessing technique. In addition, the proposed system utilized a signal smoothing filter to allow the correction of the frequency by eliminating the lowest frequency components of the system by approximately 40 Hz. Specifically, the system adopted the mean-smooth filter due to its low computational complexity and its high degree of noise removal [34]. Equation (2) represents the output of the application of the filter with a window of k=20, where $V_{i}$ represents the value of the samples taken by the ES.

(2) $$V_{out}={\left(2w+1\right)}^{-1}\sum_{i=t-w}^{t+w}V_{i}.$$

**Figure 3:**
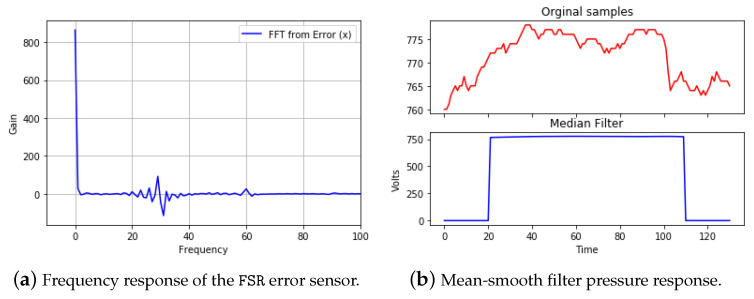
Testing results of the sensor’s filtered data.

Different pressure tests were applied in order to establish different plantar sizes. Figure 3 exemplifies, on the one hand, the frequency analysis of the FSR error sensor, and, on the other hand, the mean-smooth filter pressure response.

### 3.3. Electronic System Description

The proposed system was composed of the hardware and software coupling of 14 sensors, as well as a microprocessor to acquire the pressure data. The ATmega2560 processor was chosen due to the large number of analog converters, which enabled the integration of all sensors and the coupling. In addition, in order to have a system data interpretation interface, a communication bridge between the electronic system and a computer was required. The system used the NRF24L01 module, a 2.4 GHz-band RF transceiver that allows wireless communication to each microprocessor. This protocol was established due to its reliability over long distances, low interference, and low energy consumption. In order to allow the control of messages by several RF modules, the proposed system utilized a centralized ATmega328 processor, due to its small size and computational conditions for sending/receiving data from the RF module. For evaluation purposes, the proposed system could be connected to a computer to permit a proper data visualization through a custom graphical user interface (GUI).

The use of 14 sensors for data acquisition required the analysis of the current consumption in order to dimension the power supply. The total power consumption was computed as the sum of the consumption of every single electronic device. As a reference, and based on the components’ datasheets, the prototype used for this research consumed 40 mA per analog input of the ATmega2560; each FSR sensor consumed 1 mA; the operational amplifiers (OAs) consumed 2.8 mA each; and the RF module consumed 12.3 mA. As a consequence, a voltage source of +5V/−5V with 600 mA of minimum current was required as the power supply. In this sense, a standard DC source of 1 A and 5 V with constant output voltage met the requirements (a diode bridge rectifier and an RC filter for ripple voltage were implemented).

## 4. Data Analysis

This section presents (a) the description of the database acquisition process that was subject to the data analysis, (b) the algorithms related to the selection of the prototypes in order to implement the supervised learning algorithm within the electronic system, and (c) the supervised classification algorithms and their evaluation metrics.

### 4.1. Original Samples

The process began by creating a controlled environment to collect robust data from the children, where they could feel comfortable, and their legal representatives agreed to their participation. Subsequently, to use the device, the child had to be barefoot, and there had to be hygienic paper where the child’s footprints were in contact with the platform. The system did not collect data as long as there was no pressure on the sensors. This was observed by means of LEDs that helped to position the child correctly with the arms relaxed at the side of the body and in a straight back position. This process was monitored by the health personnel from the educational institution. Finally, the hygienic paper was replaced after every new data collection iteration. The data acquisition process began with the identification of the footprint pressure that was generated on the sensors. These data were sent to the workstation for further analysis. For the assignment of labels, each data collection was validated with the Hernández-Corvo analysis, as well as with a specialist in the area. A total of 200 volunteer children participated in this stage for each age group. Each volunteer had between four and five samples taken, which were then filtered by the software using a mean-smooth filter.

As a result, a matrix **Y** $\in R^{m\times n}$ was generated, where ***m*** represents the number of samples and ***n*** represents the number of sensors (the attributes of each sample). ***L*** $\in R^{m\times 1}$ represents the vector of labels (flat footprint, high-arch footprint, or normal footprint). For the prototype case, the values of ***m*** and ***n*** were 400 and 14, respectively, for each size. The same sample size and number of sensors were maintained for all children to reduce errors in the information processing and data analysis. Therefore, the data matrix was divided into 80% for the training set and 20% for the test set. This matrix was stored on IEEE DataPort open datasets page [35].

### 4.2. Prototype Selection

Since the computational resources of ESs are limited, the training data selection for the supervised classification algorithm directly influences the response time of the system. The prototype selection criteria (PS) allow decreasing the data used as the input, the intrinsic knowledge of the high volume of data obtained being converged. Among all the PS methods, the most prevailing method for eliminating redundant data is the condensed nearest neighbor (CNN) algorithm, since it has been computationally proven to be suitable for reducing a large number of instances at a low computational cost [36,37].

### 4.3. Classification Algorithms

There are different classification criteria that define different limits between the edges of each label (training phase). The most prevailing should be considered, which are: (i) distance, (ii) probability, (iii) following a model, and (iv) heuristics. Nevertheless, deep learning and neural networks present a supervised learning alternative (v). Consequently, algorithms representing each of the five criteria were chosen.

The *k*-nearest neighbor (k-NN) algorithm classifies a new instance that enters the system based on the Euclidean distance into the entire training base. Generally, values of k=3 and k=5 are adequate to have a high classification performance, and not to resort to computational resources (Criterion i). The Bayesian classifier (Criterion ii) obtains the probability of each class to assign a label to the incoming data. Decision support machines present different mathematical models to work in hyperplanes (kernel functions), aiming for the distance between decision edges to be larger (Criterion iii). The decision tree algorithm performs the classification, based on recursive partitioning (Criterion iv), to generate guidelines [36]. Finally, neural networks through the use of neurons and activation functions present a model in which each neuron extracts the characteristics of the studied phenomenon and seeks to assign weights to them.

**Figure 4:**
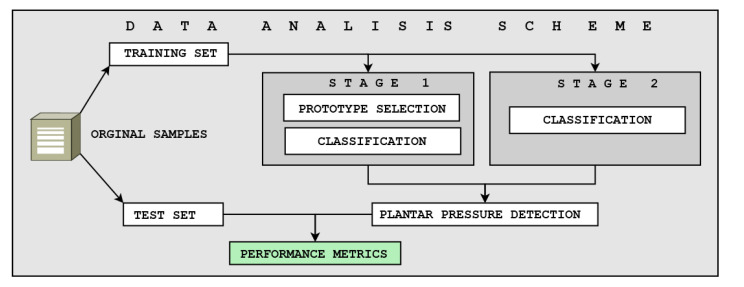
The proposed data analysis scheme. The first stage is composed of the prototype selection and classification. The second stage includes classification.

For the correct selection of the classification algorithm, two operating scenarios were proposed. The first one was based on implementing the entire solution within the electronic system using a smaller database. The second scenario focused on implementing the classification algorithm on the computer, with the ES in charge of sending the pressure data from the sensors (the entire dataset was used for this scenario). A balance measure was proposed among the response time, the classifier performance, and the internal memory consumption to select the best scenario. Furthermore, for the selection of the classifier, performance metrics based on the confusion matrix were used. This process is exemplified in Figure 4.

## 5. Results and Discussion

This section shows (a) the embedded system design, (b) the prototype selection, (c) the metrics and parameters of the machine learning algorithms, (d) the development of the display interface, and (e) the testing of the system under real operating conditions.

### 5.1. Embedded System Design

The proposed ES was deployed on a surface that could support the weight of a child (75 Kg maximum). For the installation, calibration tests were carried out with the AO and the pressure obtained by each FSR sensor.

With the aim to prove the hardware and software coupling’s effectiveness, the analysis of the signal-to-noise ratio (SNR) was conducted in three stages: (i) using the original sensor signal, (ii) with the hardware coupling, and (iii) using the software filter. It is worth mentioning that the SNR (represented in decibels) is the difference in power between the original signal and the noise inside the system. In this sense, the higher its value, the better the signal obtained is. These results are presented in Table 2.

### 5.2. Prototype Selection

In relation to Stage I, the selection of the classification algorithm was restricted to the quality of the dataset. For this reason, the prototype employed the CNN algorithm, given that it presented an enhanced performance when removing intrinsic instances that did not add knowledge to the dataset and increased the volume of the data [36]. As a result, a reduction of the data by 94.98% and 95.12% was obtained for the preschool and school children, respectively. The latter followed from an average elimination of 303 measurement cases in the training set. The simplification of the dataset led to a matrix **X** $\in R^{p\times n}$, where p=16 and n=14. Furthermore, the tag vector of **X** changed to ***K*** $\in R^{p\times 1}$. These matrices were stored in the ES’s memory.

**Figure 5:**
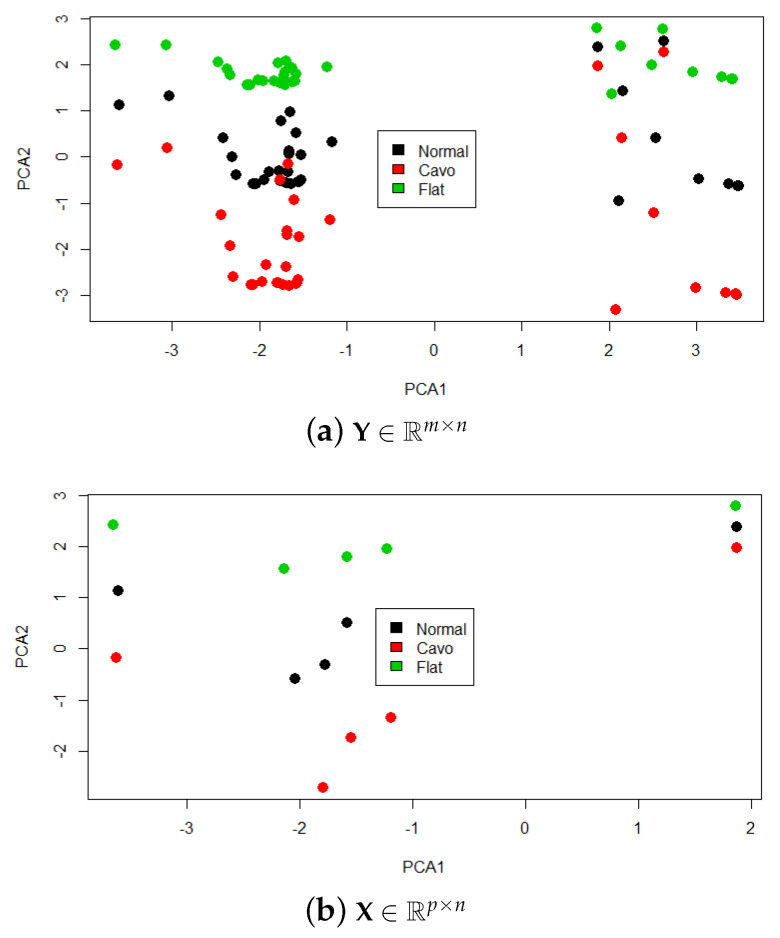
Prototype selection criterion (training set reduction).

Finally, the Y and X matrices were processed to reduce their dimensionality in order to have a graphic representation of the operation. The reduction algorithm used was principal component analysis (PCA), due to its simplicity and superior representation of high-dimensional sets in low dimensions [36]. Figure 5 shows the two-dimensional (2D) datasets, with each color representing the different types of footprints.

### 5.3. Classification Algorithms

The evaluation of each classification algorithm was based on the different metrics obtained from the confusion matrix, that is: true positive (TP), true negative (TN), false positive (FP), and false negative (FN) [38]. Each algorithm was compiled in Python, using the scikit-learn library. This process was performed using matrices Y and X. As an additional criterion for its selection, the standard parameters of each algorithm were used to verify its representation of the studied phenomenon. In this regard, *Stage II* focused on implementing the ML model without the prototype selection criterion in a GUI stored on a computer with high computational resources.

Regarding deep learning, the simplest possible model reduces the computational consumption of the neural network. The datasets had a high-dimensional data (14 dimensions). Therefore, a neural network having a single hidden layer (linear model) was not considered applicable. Consequently, it was necessary to start working with 2+ hidden layers [39]. With respect to the neurons, we expected to have a pyramidal factor to choose the smallest number of neurons; therefore, the following set of rules was considered:

(3) $$\begin{array}{c} h_{1}=\left(o*r^{2}\right), \end{array}$$

(4) $$\begin{array}{c} h_{2}=\left(o*r\right), \end{array}$$

(5) $$\begin{array}{c} r={\left(i/o\right)}^{\frac{1}{3}}, \end{array}$$

where *i* denotes the input attributes, *o* are the output variables, $h_{1}$ and $h_{2}$ stand for the minimum number of hidden layers (Numbers 1 and 2, respectively), and *r* represents the pyramidal factor. If the neuron was unable to learn in the initial settings, the number of neurons was increased until an adequate performance was reached. Experimentally, it was found that $h_{1}=10$ and $h_{2}=4$ were the optimal values for our specific ES, since a performance of 100% was not realistic.

However, our proposal was based on the premise of implementing a lightweight neural network. For this, we adopted the criterion of using a self-organizing map (SOM) architecture with a single functional layer that mapped the spatial distribution of the fourteen sensors of 10 × 10 dimensions with an activation function of a Gaussian-type neighborhood. In this context, the same results were obtained with a neural network with two hidden layers, but the SOM results used less computational power. However, for an implementation within an ES, the problem remained computationally complex.

Finally, and to fulfill the cross-validation, the dataset was randomly divided into training and testing 10 times and shuffled after every iteration. The average results are given in Table 3 and Table 4.

With the performance result of the classifier, additional parameters such as the learning speed, the classification speed, and the memory consumption can be computed [40]. According to the proposed stages (I and II), the selection of the best set of hyperparameters was performed for each stage. Taking into account that the first stage was based on the implementation of a machine learning routine in the ES and the second stage was focused on the implementation of a dedicated workstation (where the display interface was connected), the simulation results are shown in Table 5 and Table 6, respectively.

From the table results, the best supervised machine learning algorithm performance in the ATmega2560 ES (Stage I) was obtained with the *k*-NN algorithm, which gave the best classification accuracy, using the training matrix with few instances. Furthermore, the learning and classification speed of *k*-NN outperformed the algorithms based on decision trees and SVM, making the latter unacceptable solutions due to their high computational cost when using complex and multi-iteration mathematical functions, making the system convoluted when compiling the whole process. Regarding the option of training the machine learning model in the dedicated workstation (Stage II), neural networks and SVM presented high classification performances, but compromised on the learning speed. Therefore, the decision tree method, having an adequate computational capacity, had a tolerable tradeoff between the performance (100%) and classification speed. This phenomenon was explained as follows: once the model finished iterating, the results were presented with simple constraints to execute.

### 5.4. Data Visualization

**Figure 6:**
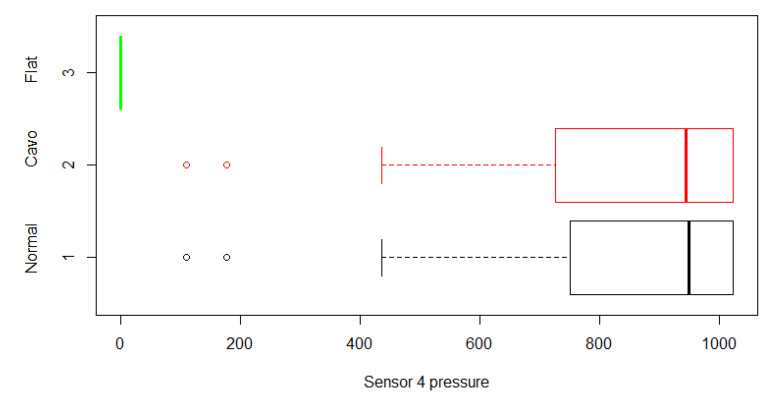
Normal distribution analysis of Sensor 4.

The data visualization step was inspired by the software “Processing”, since it was designed for the interaction between ESs and a computer. The design of the interface was based on the representation of the location of each sensor on a footprint, showing the pressure levels generated by the user. The classification inference of the system could be observed graphically, transforming the character string of the pressure levels of each of the sensors into readable content. The analysis of the standard probability distribution led us to determine the pressure values in relation to the type of footprint. In Figure 6, the values of Sensor Number 4 are presented with different colors on the interface, as well as the digital range that the system obtained when this specific sensor was activated. In the last case, if the generated pressure did not cause a digital value of 200 mV in the analog–digital converter of the ES, it was not represented on the interface. At the same time, if the pressure value was between 200 mV and 400 mV, it would be shown as green since it represented the slight pressure of the foot on the surface containing the sensor. If the value was in the range of 400 mV to 800 mV, the interface would change to orange, representing a medium pressure reading. Finally, if the sensor value was higher than 800 mV, the color would be red since the sensor was fully activated.

**Figure 7:**
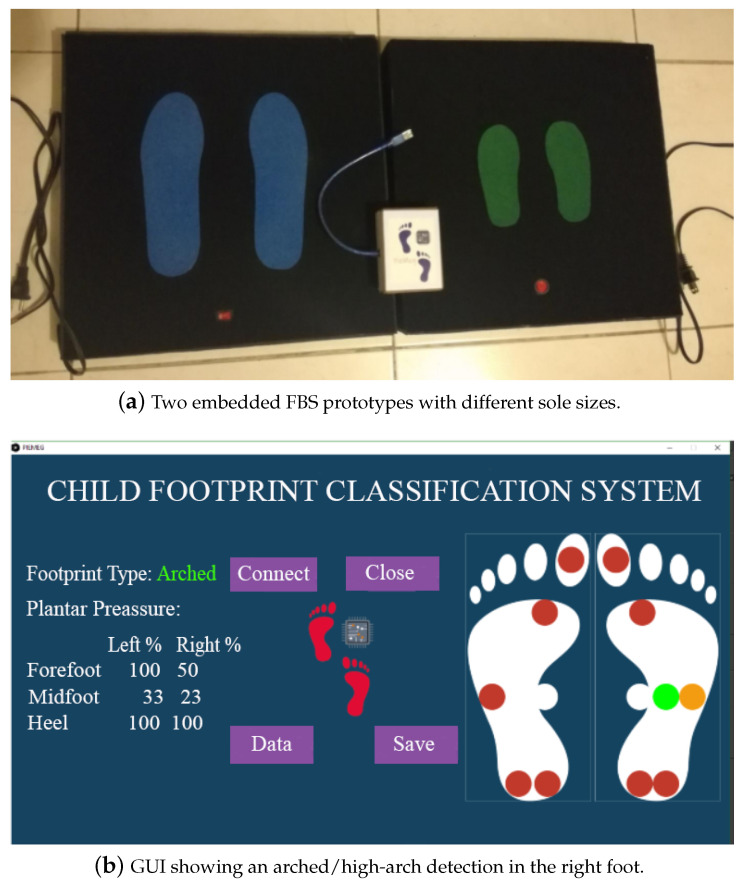
Hardware and software prototype used for the data collection stage. (**a**) The embedded sensor testbed with hardware coupling; (**b**) visualization and analysis of the plantar pressure points on the graphical interface.

The GUI presented additional options for: (a) registering the personal information of each user, (b) recording the plantar pressure data per user, (c) recording the pressure of each individual sensor, and (d) recording the classification output of the algorithm. An example of its operation is shown in Figure 7.

### 5.5. System Implementation with the Real Test

With the two learning stages established and their respective algorithms, the tests were conducted under a controlled environment together with specialist doctors, who validated the results of our system by using the *Hernández Corvo* methodology. After the system acquired, recorded, and classified the pressure points by color according to the children’s feet, the system assigned a footprint type and stored the values in the system’s memory. Subsequently, the child covered his/her feet with talcum powder and stepped on a black piece of cardboard so the doctors could manually analyze and validate the footprint to estimate the accuracy of the system. As a result, our system attained a performance of 97% in the software tests. It should be noted that we selected a balanced group of volunteers with footprint sizes within the minimum and maximum established dimensions.

**Figure 8:**
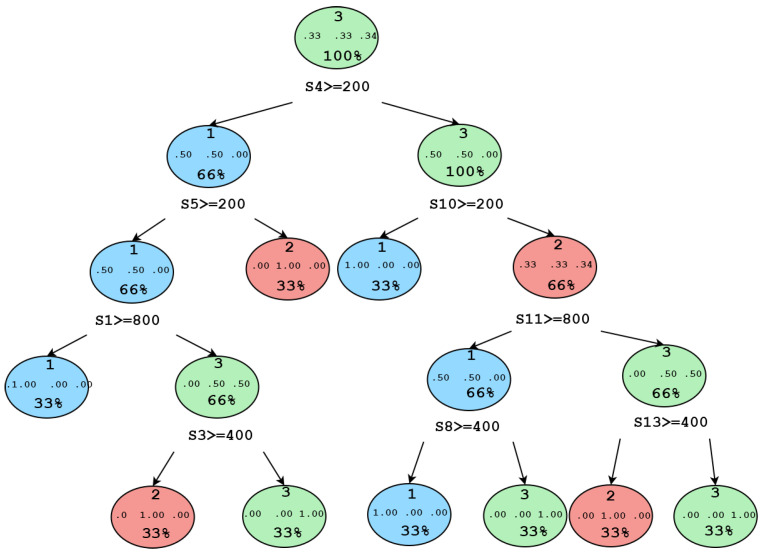
Conditional hierarchy used by the decision tree algorithm.

We discovered that implementing the algorithm inside the workstation where the interface was installed caused the performance to be volatile. On further analysis, we detected that a performance increase of the decision tree algorithm was possible by hierarchically feeding the sensor data to the model. We also detected failures when the volunteers did not position their foot correctly over the sensors. The conditional diagram of the algorithm is shown in Figure 8.

From the data analysis of Stage I, the *k*-NN algorithm was developed within the electronic system with the reduced data matrix **X**. As a result, it was observed that *k*-NN maintained a high performance (97.2%), since the algorithm related a new footprint to the most similar ones within the training set. Therefore, the *k*-NN algorithm maintained its simulated performance despite having the same conditions as in Stage II. For this reason, our research group decided to implement this criterion in the final system build.

**Figure 9:**
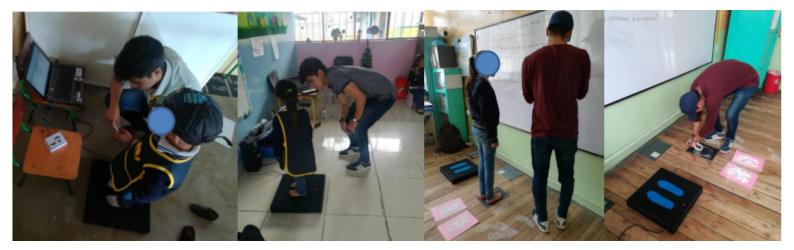
Program volunteers during the acquisition of the real data. The embedded FBS was operated under a controlled environment.

With the final system, operational tests were implemented at several educational institutions in order to determine the type of footprint among the previously established types of volunteers. This process was performed in the province of Imbabura-Ecuador, where a total of 1000 preschool children and 600 school children participated in this research. Figure 9 shows the use of the platform at the academic institutions without the support of other footprint detection techniques.

Table 7 summarizes the results obtained in the operation tests of the system. It can be observed that 73% of preschool and 56% of school children had problematic footprints. However, it can be seen that the percentage decreased toward the end of childhood. This was mainly due to the changes in their physical activities, when performing more exercises related to specific sports. In other cases, the percentage of the flat feet was evident, and the children were provided specific footwear to correct their footprints [7,41]. It should be emphasized that when asking the volunteers if they had undergone a pediatric footprint analysis, only 15% responded positively. The statistical results of the in situ tests are shown in Table 7.

The proposed system proved to be an effective approach to prevent future health problems in children up to 12 years old that were developing an abnormal footprint. Physiotherapy sessions were granted to the children developing foot anomalies in order to correct their footprint. On the other hand, when dealing with children, the data collection effectiveness was directly proportional to the degree of attention to the instructions for the use of the prototype. For this reason, the implementation of algorithms based on rules caused a decrease in the classification performance. Finally, the main objective of this research was to provide an easy-to-use tool for the rural sector of Ecuador, where, in many cases, parents do not have the economic resources for their children’s medical and preventative care.

## 6. Conclusions and Future Works

We present our conclusions in relation to: (i) the embedded system design, (ii) the data analysis, and (iii) the feasibility of the system in real conditions:

(i) The proposed ES fulfilled our expectations regarding its functionality. This was due to an adequate coupling and filtering of the data, both in the hardware and software. As a consequence, the analysis of the noise components and the implementation of the active electronic elements guaranteed that the data acquisition process was adequate to represent the studied phenomenon;
(ii) The analysis scheme presented in this work had the option of implementing the supervised classification algorithm in the ES or in the dedicated workstation with the GUI installed. As a result, it was proven that in the simulation, the decision tree algorithm performed adequately; under real-world conditions, the performance was far that expected. For this reason, the *k*-NN algorithm was selected, with a kernel value of k=3 as the optimal alternative. In addition, we decided to reduce the training set by preprocessing using the CNN algorithm, which is strongly recommended if these types of solutions are deployed. Finally, the field tests performed in relation to the metrics of the classification algorithm and their selection parameters were essential to achieve the expected classification accuracy;
(iii) Regarding the tests of the system in real conditions, on the one hand, we compared the classification algorithm output with the *Hernández Corvo* method to validate the functionality. For this reason, we propose follow-up studies to detect abnormalities in the footprint and alert parents to seek early foot correction for their children. Furthermore, it is expected that rural health centers will replicate the prototype to enable an early detection of children’s plantar problems, since the proposed prototype was a low-investment, portable/mobile, and high-performance system. It is important to point out that this research effort sought to generate a prognosis of the child’s footprint, but did not intend in any case to replace a visit to a specialist in the area who can confirm the problem and provide the appropriate diagnosis and treatment.

As future works, we plan to design a system that will include the majority of foot sizes present in the Ecuadorian population, generate a more general dataset, and enable an efficient early detection of plantar problems, not only in children, but also in adults with respect to the average type of footprint.

## Figures and Tables

**Table 1:** Highest plantar zone pressure by footprint type.

Footprint Type	Highest Plantar Zone Pressure
Normal foot Label 1	- First metatarsal of the big toe - Middle half zone - Heel
Arched/high-arch foot Label 2	- First metatarsal of the big toe - Right side midfoot area - Average heel region
Flat foot Label 3	- Union between the big toe and second toe - Medial distal point of the midfoot - Midheel area

**Table 2:** Signal type vs. SNR outcome.

Signal	SNR Outcome (dB)
Original signal	3.25
Hardware coupling	4.12
Software filter	6.23

**Table 3:** Matrix X classification metrics’ comparison under Stage I.

Comparison Metrics	k-NN	Naive	Decision	SVM
(%)	k=3	Bayes	Tree	(Sigmoid)
	Normal Footprint (μ)			
**Accuracy (%)**	**98.7%**	61.7	30	97.5
**Error rate (%)**	**1.2%**	38.2	70	2.4
**Sensitivity (%)**	**100%**	58.8	30	100
**Specificity (%)**	**98.1%**	63.8	0	96.3
**Precision (%)**	96	54	**100%**	92.8
**Recall (%)**	32.5	40	NN	**49%**
**Geometric mean (%)**	**37.46%**	24.49	0	37.12
	**Flat Footprint (μ)**			
**Accuracy (%)**	**98.7%**	61.7	30	97.5
**Error rate(%)**	**1.2%**	38.2	70	2.4
**Sensitivity (%)**	**100%**	17.6	0	**100%**
**Specificity (%)**	66.2	73.4	**100%**	96.3
**Precision(%)**	**96%**	15	0	90.8
**Recall (%)**	33.7	6	0	**49%**
**Geometric mean (%)**	**38%**	11.87	0	37.1
	**High-Arch Footprint (μ)**			
**Accuracy (%)**	**98.7%**	61.7	30	97.5
**Error rate (%)**	**1.2%**	38.2	70	2.4
**Sensitivity (%)**	**100%**	65.8	0	**100%**
**Specificity (%)**	66.2	57.5	**100%**	96.3
**Precision (%)**	**96.4**	61.3	0	90.8
**Recall(%)**	33.7	**54%**	0	49
**Geometric mean (%)**	**38.1%**	24.9	0	37.1

**Table 4:** Matrix Y classification metrics’ comparison under Stage II.

Comparison Metrics	k-NN	Naive	Decision	SVM	Neural
	k=3	Bayes	Tree	(Sigmoid)	Network
	Normal Footprint (μ)				
**Accurac**	98.7%	76.5%	**100%**	97.5%	**100%**
**Error rate**	1.2%	23.4%	**0%**	2.4%	**0%**
**Sensitivity**	**100%**	72.4%	**100%**	**100%**	**100%**
**Specificity**	98.1%	78.8%	**100%**	96.3%	**100%**
**Precision**	96%	65.6%	**100%**	90.8%	**100%**
**Recall**	32.5%	33.8%	**50%**	49%	**50%**
**Geometric mean**	37.4%	29.34%	38.1%	37.1%	**38.1%**
	**Flat Footprint (μ)**				
**Accuracy**	98.7%	76.5%	**100%**	97.5%	**100%**
**Error rate**	1.2%	23.4%	**0%**	2.4%	**0%**
**Sensitivity**	**100%**	58.3%	**100%**	**100%**	**100%**
**Specificity**	66.2%	81.3%	**100%**	96.3%	**100%**
**Precision**	96.4%	56%	**100%**	90.8%	**100%**
**Recall**	33.7%	22.5%	**50%**	49%	**50%**
**Geometric mean**	**38.1%**	25.92%	**38.1%**	37.1%	**38.1%**
	**High-Arch Footprint (μ)**				
**Accuracy**	98.7%	76.5%	**100%**	97.5%	**96%**
**Error rate**	1.2%	23.4%	**0%**	2.4%	**4%**
**Sensitivity**	**100%**	77.1%	**100%**	**100%**	**96%**
**Specificity**	66.2%	76%	**100%**	96.3%	**100%**
**Precision**	96.4%	7.10%	0%	90.8%	**96%**
**Recall**	33.7%	**77.1%**	50%	49%	50%
**Geometric mean**	**38.1%**	30.74%	**38.1%**	37.1%	**38.1%**

**Table 5:** Classification algorithms comparative parameters’ study under Stage I.

Comparison Parameters	k-NN k=3	Naive Bayes	Decision Tree	SVM (Sigmoid)
**Learning speed**	Average	Average	Worst	Worst
**Classification speed**	Average	Average	**Best**	Worst
**Performance**	**Best**	Average	Worst	**Best**
**Memory size**	Average	Average	Worst	Worst

**Table 6:** Classification algorithms comparative parameters’ study under Stage II.

Comparison	k-NN	Naive	Decision	SVM	Neural
Parameters	*k* = 3	Bayes	Tree	Sigmoid	Network
**Learning speed**	Average	Best	Average	Worst	Worst
**Classification speed**	Average	Average	**Best**	Worst	Average
**Performance**	Average	Average	**Best**	Average	**Best**
**Memory size**	Average	Average	Average	Average	Worst

**Table 7:** Footprint analysis of pediatric patients.

Pediatric Patient	Footprint Type		
	Normal	Flat	High Arch
Preschool	27%	63%	10%
School-age	44%	52%	4%

## Data Availability

Dataset is available in [35].
